# Elastic Die Technology for Spur Gear Powder Compaction: Experimental Measurements and Simulation-Based Validation

**DOI:** 10.3390/ma19061203

**Published:** 2026-03-19

**Authors:** Dan Cristian Noveanu

**Affiliations:** 1 Materials Science and Engineering Department, Technical University of Cluj-Napoca, 103–105 Muncii Avenue, 400641 Cluj-Napoca, Romania; dan.noveanu@ipm.utcluj.ro; 2 EUT+ European University of Technology, European Union

**Keywords:** metal powder compaction, elastic die, finite element analysis

## Abstract

Achieving high density in complex powder metallurgy components like spur gears is often hindered by friction-induced density gradients and ejection defects. This study investigates a novel elastic die system designed to mitigate these issues through controlled radial deformation. Spur gears were compacted using Ancorsteel 2000 powder under pressures of 400–700 MPa, utilizing a tapered elastic sleeve to apply radial compression. Green and sintered densities were measured, while porosity distribution was quantified via image analysis. Additionally, a 3D finite element simulation using FORGE software was conducted to model the thermo-mechanical behavior and stress distribution during the process. Experimental trials demonstrated that the elastic relaxation of the sleeve enabled free ejection of the compacts without requiring an extraction force. Image analysis confirmed a homogenous porosity distribution across the gear teeth, and higher die pre-stressing strokes were found to correlate with increased sintered density. Finite element modeling accurately predicted critical stress concentrations of 700 MPa at the die–sleeve interface and validated the strain distribution. The results confirm that elastic die technology effectively eliminates ejection friction and improves density uniformity in complex gears, offering a viable solution for reducing tool wear and manufacturing defects in high-precision powder metallurgy.

## 1. Introduction

Powder metallurgy (PM) is a well-established manufacturing process widely utilized for producing components with high dimensional precision and material efficiency. A primary objective in modern PM is achieving high density, as this property is directly correlated with the mechanical performance and reliability of the final component [1,2,3,4]. However, as the relative density of a part approaches the theoretical limit, significant challenges arise when using conventional rigid dies, particularly for components with complex geometries [5,6,7,8].

In standard rigid die compaction, the applied pressure is not distributed hydrostatically. Friction between powder particles and the die walls leads to density gradients, often resulting in lower densities in the neutral zones of the compact [9]. Furthermore, the ejection phase is critical; as the extraction force increases with density, the differential elastic “spring-back” of the part upon exiting the die can generate shear stresses [10]. These stresses frequently lead to defects such as laminar cracks, flaking, or capping, especially in complex-shaped components. Additionally, the high friction forces involved in forcibly ejecting high-density parts significantly accelerate wear on the active surfaces of the tooling.

To address these limitations—specifically the density non-uniformity and ejection defects—elastic die technology has emerged as a viable alternative solution [11]. Unlike rigid tools, this technology uses a deformable element (sleeve) to manage residual stresses. The concept relies on an elastic sleeve with a tapered external surface housed within a rigid container. During compaction, the sleeve is forced into the container, reducing its radial dimensions and exerting lateral pressure on the powder, simulating isostatic pressing conditions [12,13]. The most distinct advantage of this method is the “free ejection” phenomenon. Upon removal of the pressing force, the elastic relaxation (expansion) of the sleeve exceeds the spring-back of the metal powder compact, effectively detaching the die walls from the part before ejection begins. This eliminates extraction friction, thereby preventing cracks and extending tool life [14].

While the elastic die concept has been theoretically explored, its practical application to complex geometries like gears requires rigorous validation [15]. The purpose of this study is to investigate the compaction of spur gears using Ancorsteel 2000 low-alloy steel powder within an elastic die setup. This work aims to experimentally determine the influence of lubricant content and die pre-stressing on the density and porosity distribution of the gears. Furthermore, to validate the experimental results and better understand the internal mechanics, a 3D Finite Element Method (FEM) simulation is performed using FORGE 2009 software.

The novelty of this study lies in the practical validation of the elastic die concept for a highly complex industrial geometry—a multi-tooth spur gear—using high-performance Ancorsteel 2000 powder, which has not been comprehensively documented in the existing literature. Furthermore, this study innovates by pairing experimental porosity quantification (to prove isostatic-like densification) with a 3D thermo-mechanical finite element simulation to identify the precise fatigue bottlenecks in the elastic tooling, thereby providing a complete framework for industrial implementation.

The principal conclusions of this work demonstrate that the elastic die technology successfully allows for the free ejection of spur gears without extraction force. Image analysis confirms a homogeneous porosity distribution across the gear teeth, and the FEM simulation accurately predicts the strain fields and critical stress concentrations at the tool interface, validating the technology for high-precision manufacturing.

## 2. Materials and Methods

### 2.1. Materials

The material selected for the experimental investigation of spur gear compaction was Ancorsteel 2000 [16], a water-atomized low-alloy steel powder produced by Hoeganaes Corporation (Cinnaminson, NJ, USA). This powder is specifically designed for high-performance structural applications requiring high density and strength. The chemical composition includes Nickel (0.46%), Molybdenum (0.61%), Manganese (0.25%) and Oxygen (0.13%), with the balance being Iron [17].

To facilitate the compaction process and minimize friction between the powder particles and the die walls, Zinc Stearate Zn(C_18_H_35_O_2_)_2_ was used as a solid lubricant and binder. The lubricant was mechanically mixed with the metal powder in two distinct concentrations to evaluate its influence on compressibility and ejection: 0.5 wt.% Zinc Stearate and 0.75 wt.% Zinc Stearate.

### 2.2. Specimen Geometry

**Figure 1:**
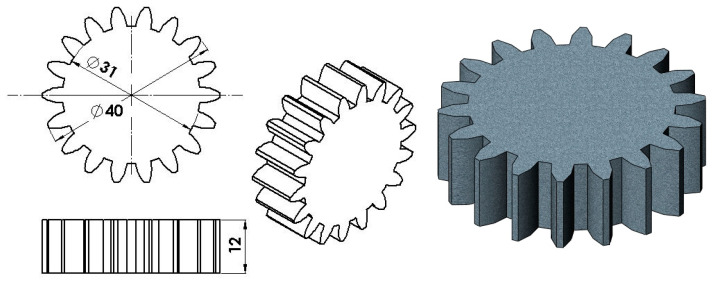
Geometry and dimensions of the spur gear specimen.

The experimental study focused on producing a spur gear with complex geometry. The specific dimensions of the gear, featuring z = 18 teeth and a module of 2 mm, are illustrated in Figure 1.

### 2.3. Experimental Setup and Die Design

**Figure 2:**
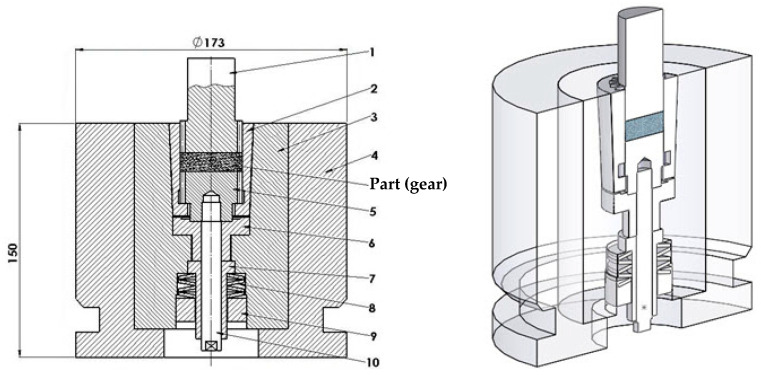
Schematic cross-section of the elastic die assembly: 1—punch; 2—elastic sleeve; 3—container; 4—die-holder block; 5—lower die; 6—bushing; 7—guide bushing; 8—disc spring; 9—ring; 10—extractor rod.

A specialized elastic die assembly was designed and manufactured to produce a spur gear geometry with 18 teeth. The assembly, illustrated in Figure 2, consists of four main components:

1. Elastic Sleeve: A deformable element with a cylindrical interior profiled to the negative geometry of the gear teeth and a tapered conical exterior (machined from C120 high-carbon tool steel, selected for its high elastic limit and excellent wear resistance).
2. Container: A rigid outer ring (die body) featuring an internal taper that matches the exterior of the elastic sleeve (machined from C45 medium-carbon steel to provide necessary structural toughness).
3. Punch and Extractor: Rigid tools profiled to match the gear teeth (made of C120 tool steel for high compressive strength).
4. Die-Holder Block: Supports the assembly components (made of C45 Steel).

Operating Principle: The elastic sleeve is mounted within the container with the capability to execute a controlled vertical translational movement. The process utilizes single-action press kinematics. During the pressing stroke, the vertical displacement of the elastic sleeve is not independently actuated by a secondary hydraulic ram. Instead, it is driven by the downward force transmitted through the upper punch and the friction between the compacted powder mass and the inner walls of the sleeve. This combined force pushes the sleeve axially into the tapered container against the resistance of the underlying disc springs. Due to the wedge effect of the conical contact surfaces (3° taper angle), the sleeve undergoes a controlled radial contraction (ΔD). This action exerts lateral pressure on the powder, compressing it radially while the punch compresses it axially, thereby mimicking isostatic pressing conditions.

Free Ejection Mechanism: Upon the removal of the pressing force, the sleeve moves vertically in the opposite direction, assisted by disc springs. Due to its inherent elasticity, the sleeve expands radially, returning to its initial diameter. This elastic relaxation is designed to be greater than the elastic spring-back of the compacted powder part. Consequently, the die walls detach from the component, allowing the extractor to lift the gear without friction forces, achieving “free ejection.”

**Figure 3:**
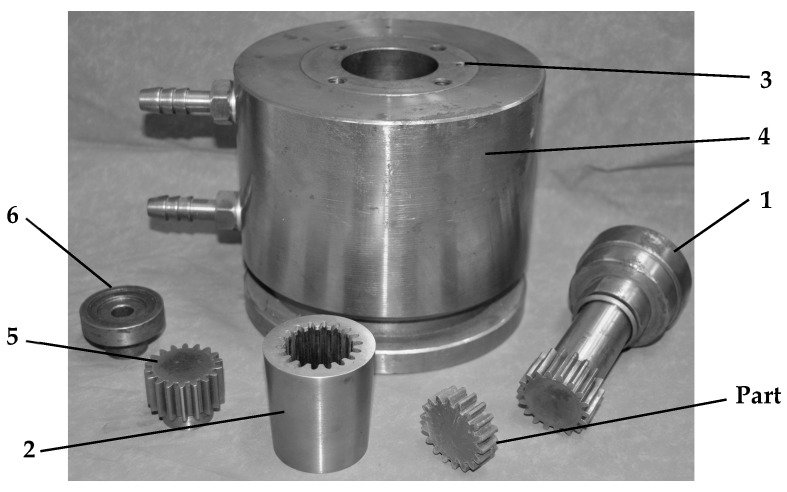
The main elements of the elastic die: 1—punch; 2—elastic sleeve; 3—container; 4—die-holder block; 5—lower die; 6—bushing.

The physical components realized for this experiment are displayed in Figure 3.

### 2.4. Experimental Protocol

**Figure 4:**
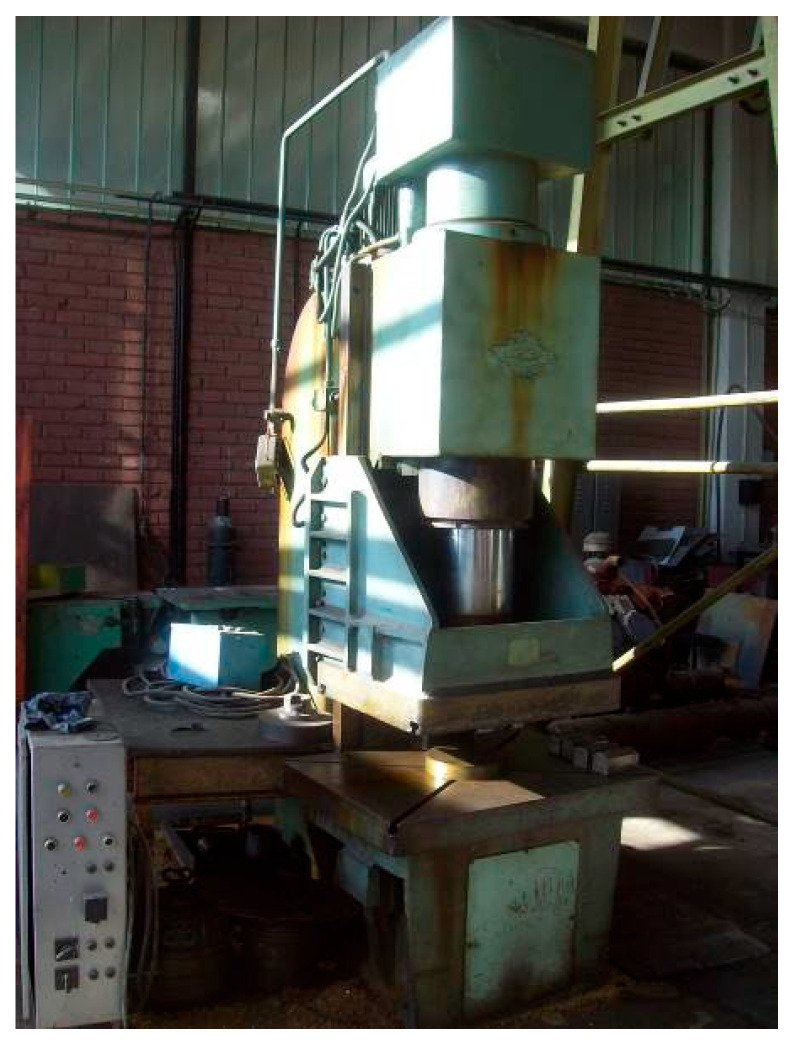
PH150 Hydraulic Press.

The compaction trials were conducted using a single-action PH150 Hydraulic Press, Intreprinderea Mecanica Sibiu, Mârșa, Sibiu County, Romania, 1977 (Figure 4) with a maximum force capacity of 150 kN, located at the Metal Forming Laboratory of the Technical University of Cluj-Napoca.

The experimental design involved varying three primary parameters:

1 Compaction Pressure: Samples were pressed at 400, 500, 600, and 700 MPa (N/mm^2^).
2 Die Pre-stressing (Sleeve Stroke): To evaluate the effect of radial compression, three pre-stressing conditions were tested:
  - No pre-stressing.
  - Die stroke of 2 mm.
  - Die stroke of 3 mm.
3 Lubricant Content: 0.5% and 0.75% mixtures.

Following compaction, the green parts were sintered in an industrial belt furnace at S.C. Sinterom S.A. (Cluj-Napoca, Romania). The sintering process was conducted at a temperature of 1120 °C.

### 2.5. Characterization Methods

The density of the components was determined using the Archimedes method. Samples were weighed in air and subsequently in water to calculate both green density and sintered density.

Microstructural analysis was performed using optical microscopy. To quantify the porosity distribution, cross-sectional images were processed using ImageJ 1.53 v. 21, an open-source image processing software. Micrographs were binarized to distinguish between the metallic matrix and pores. Measurements were taken in two critical regions:

- Central Area: Upper, median, and lower sections of the gear axis.
- Tooth Area: Root, median, and tip of the gear teeth.

Due to the intensive loads and the experimental nature of the specialized tooling, single specimens were compacted for each unique parameter configuration. To account for experimental variability, the measurement uncertainty associated with the Archimedes density determination method was calculated as ±0.04 g/cm^3^. This standard error is incorporated into the graphical representation of the density results to provide a reliable margin of statistical confidence.

### 2.6. Numerical Simulation Setup

To analyze stress distributions at the tool-part interface, a 3D Finite Element Method (FEM) simulation was performed using FORGE 2009 software (Transvalor).

The material behavior was modeled using a Norton–Hoff viscoplastic law [18,19,20], suitable for simulating large deformations. The deviatoric stress tensor is defined as

(1) $$s=2A(T,\overset{-}{\varepsilon },\ldots )(\sqrt{3}\cdot \overset{\overset{.}{-}}{\varepsilon }{)}^{m-1}\overset{•}{\varepsilon }$$

where *s* is the stress tensor deviator, and *A*(*T*, …) is the consistency or material strength. This is not a fixed number; it is a function of temperature (*T*), equivalent strain ($\overset{-}{\varepsilon }$), and other microstructural variables [mm·Kg·s], *m* [-]—the strain rate sensitivity index, $\overset{\overset{.}{-}}{\varepsilon }$—the equivalent (scalar) strain rate and $\overset{•}{\varepsilon }$ [s^−1^]—strain rate tensor. The rheological behavior was defined using the Hansel-Spittel equation to account for temperature and strain rate sensitivity:

(2) $$\sigma =A\cdot e^{m_{1}T}\cdot T^{m_{9}}\cdot \varepsilon^{m_{2}}\cdot e^{m_{4}/\varepsilon }\cdot (1+\varepsilon {)}^{m_{5}\cdot T}\cdot e^{m_{7}\cdot \varepsilon }\cdot {\overset{•}{\varepsilon }}^{m_{3}}\cdot {\overset{•}{\varepsilon }}^{m_{8}T}$$

where *σ* is the flow stress, *A* is the consistency of the solid [mm·Kg·s], *T* is the temperature, ε is the plastic strain (degree of deformation), $\overset{•}{\varepsilon }$ is the strain rate (speed of deformation), and m_1_ through m_9_ regression are coefficients specific to the material being modeled. The material modeled was an Ancorsteel 2000 steel equivalent. It should be noted that FORGE software, designed for bulk metal forming, does not include constitutive models for powder compaction densification [21,22] (which requires porous plasticity models accounting for density evolution). Therefore, this simulation models the powder compact as an equivalent fully dense solid material. The primary objective is to analyze stress concentrations and strain distributions in the tooling components (elastic sleeve, container, punches) and to provide a qualitative assessment of deformation patterns in the part geometry. The simulation does not predict powder densification, porosity evolution, or absolute density values.

Boundary Conditions and Discretization:

- Friction: A Coulomb friction model was applied with a coefficient of µ = 0.3 for all contact interfaces. This value was selected based on established literature data for high-pressure forming of lubricated steel components, and it is consistent with the standard default parameters recommended by the FORGE software for such thermo-mechanical conditions.
- Kinematics: The hydraulic press speed was set to 30 mm/s.
- Meshing: The deformable bodies (Part, Elastic Sleeve, Container) were discretized using tetrahedral elements. The final mesh consisted of 182,387 nodes and 850,285 elements, with refined meshing at the contact surfaces to ensure computational accuracy (See Table 1).

**Figure 5:**
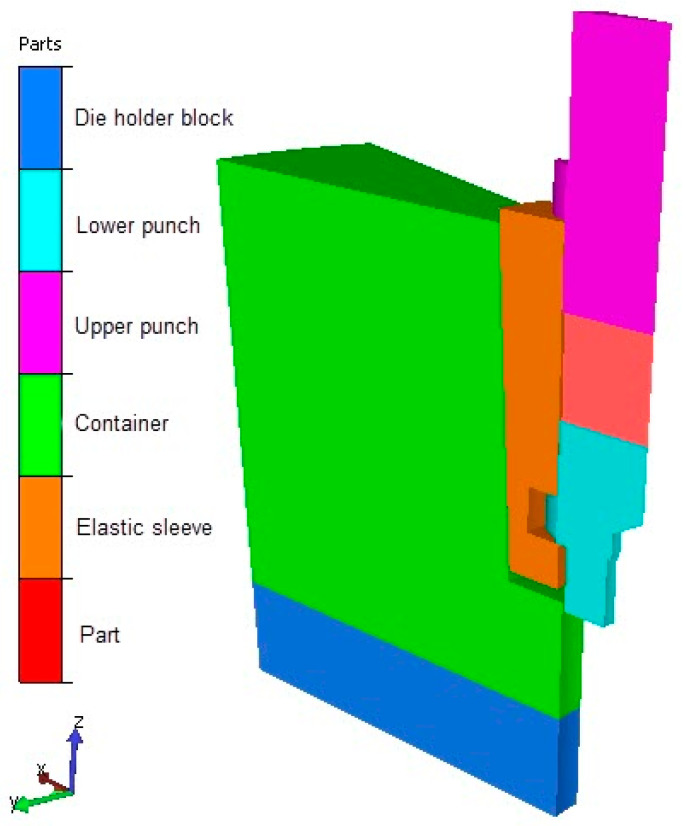
Three-dimensional Geometry of a sector of the elastic die assembly used in FORGE simulation.

The geometry used for numerical analysis, representing a sector of the gear and die assembly, is shown in Figure 5.

Data was recorded incrementally every 0.2 mm of the punch stroke. The parameters of the Hansel–Spittel behavior model Equation (2) generated by the calculation code are shown in Table 2.

## 3. Results

The performance of the elastic die was evaluated through three key experimental indicators: the influence of process parameters on density (green and sintered), the achievement of uniform porosity in complex zones, and the elimination of extraction forces during ejection.

### 3.1. Density Evolution

The density of the spur gears was measured under varying compaction pressures (400–700 MPa), lubricant contents (0.5% vs. 0.75%), and die pre-stressing conditions (sleeve strokes of 0 mm, 2 mm and 3 mm) (Table 3).

**Figure 6:**
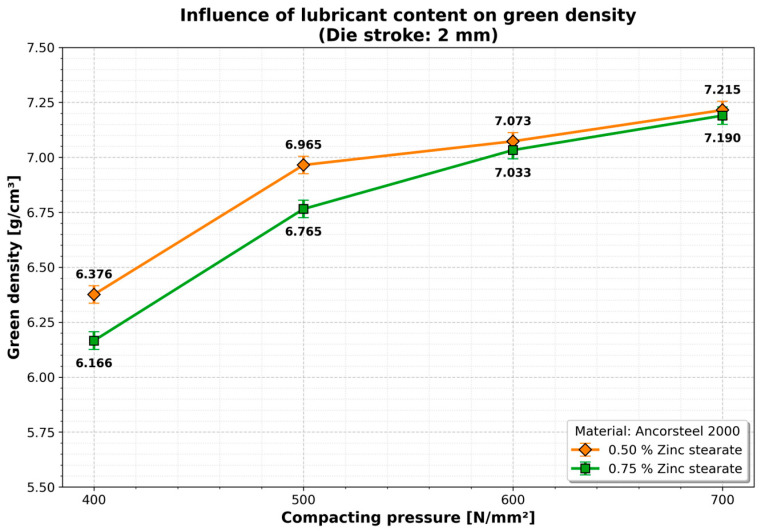
Influence of lubricant content on green density (Die stroke: 2 mm). Data points represent single-specimen measurements for each condition. Error bars indicate the estimated measurement uncertainty (±0.04 g/cm^3^) associated with the Archimedes method.

The influence of the lubricant was particularly pronounced at lower compaction pressures. As shown in Figure 6 (for a 2 mm die stroke), increasing the lubricant content from 0.5% to 0.75% resulted in a density increase of approximately 9.0% to 9.9% in the 400–500 MPa range.

However, at higher pressures (600–700 MPa), this influence diminished, with gains saturating between 3.1% and 6.9%. This indicates that while lubricant aids particle rearrangement at low loads, mechanical interlocking dominates at high loads.

**Figure 7:**
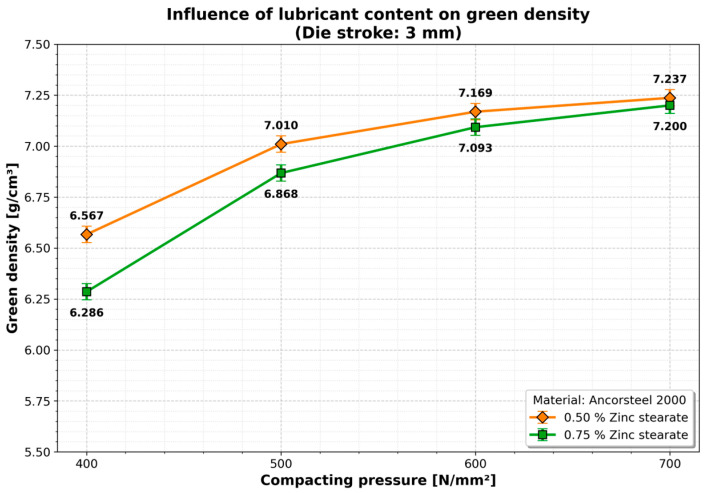
Influence of lubricant content on green density (Die stroke: 3 mm). Data points represent single-specimen measurements for each condition. Error bars indicate the estimated measurement uncertainty (±0.04 g/cm^3^) associated with the Archimedes method.

A similar trend was observed for the 3 mm die stroke (Figure 7), where higher compaction pressures yielded diminishing returns regarding lubricant addition.

**Figure 8:**
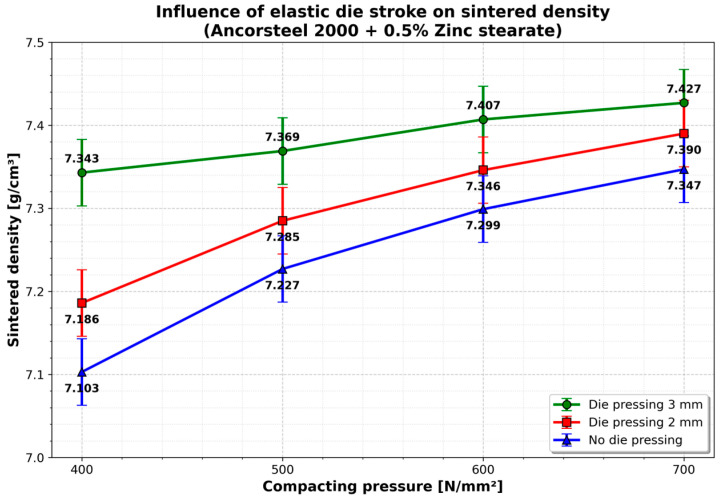
Influence of elastic die stroke on sintered density (Ancorsteel 2000 + 0.5% Zinc stearate). The highest density was achieved with a 3 mm pre-stressing stroke. Error bars indicate the estimated measurement uncertainty (±0.04 g/cm^3^) associated with the Archimedes method.

Sintering at 1120 °C resulted in a consistent increase in density across all samples. A critical finding regarding the elastic die mechanics is the influence of the pre-stressing stroke. As illustrated in Figure 8, applying a pre-stress to the sleeve significantly improved the final density compared to non-prestressed samples.

Increasing the stroke from 2 mm to 3 mm yielded higher densities, but the percentage difference was relatively narrow (3.0–3.41%).

The influence of the lubricant on the green density is important at low compaction pressures (400–500 MPa), where a linear evolution is observed, with increases between 9.0% (0.5% zinc stearate) and 9.9% (0.75% zinc stearate), with a die stroke of 2 mm (Figure 7). At higher compaction pressures (500–700 MPa) the density increase is lower reaching 6.9% (0.5% zinc stearate) and 3.1% (0.75% zinc stearate). The quantitative influence of the lubricant becomes insignificant at higher compaction pressures (600–700 MPa).

A significant change in the density obtained after sintering is observed in samples pressed with 0.5% zinc stearate (an increase of 4.4%). The density values determined after sintering at different stages of compaction, evidenced by the punch stroke, show a constant upward trend with a similar evolution. This suggests an optimal operating window where increasing radial compression beyond a certain threshold yields diminishing returns in densification.

### 3.2. Microstructural Analysis and Porosity Distribution

To verify the homogeneity of the gears—a common challenge in rigid die compaction where neutral zones often exhibit high porosity—microstructural analysis was performed. The microstructural and porosity analyses presented in this section were conducted on spur gear specimens compacted under the parameters that yielded the highest densification: a compaction pressure of 700 MPa, an elastic die pre-stressing stroke of 3 mm, and a lubricant content of 0.5 wt.% Zinc Stearate. Cross-sections were analyzed using ImageJ software to quantify porosity in two critical zones: the Axis and the Gear Teeth.

**Figure 9:**
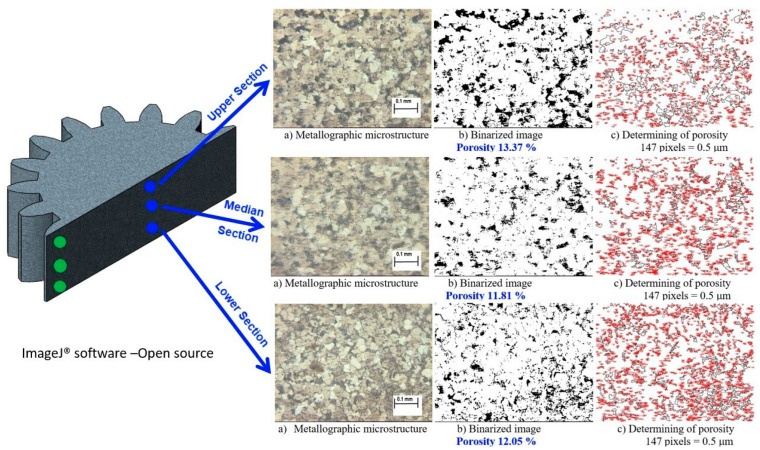
Porosity analysis in the Central Area: (**a**) metallographic microstructure; (**b**) binarized image; (**c**) porosity calculation.

Central Area Porosity (Figure 9): The analysis revealed a highly uniform distribution along the height of the gear axis:

- Lower Section: Porosity of 12.05%.
- Median Section: Porosity of 11.81%.
- Upper Section: Porosity of 13.37%.

**Figure 10:**
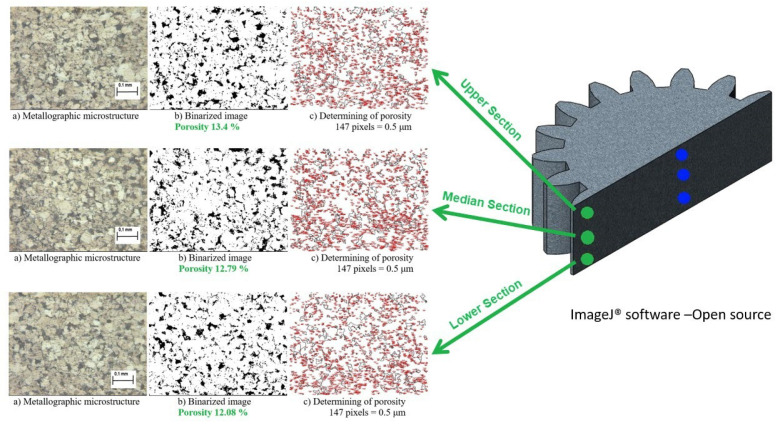
Porosity analysis in the Tooth Area: (**a**) metallographic microstructure; (**b**) binarized image; (**c**) porosity calculation.

Tooth Area Porosity (Figure 10): The complex geometry of the teeth typically concentrates stress and density gradients.

However, the elastic die achieved remarkable uniformity in this region as well:

- Lower Section: Porosity of 12.08%.
- Median Section: Porosity of 12.79%.
- Upper Section: Porosity of 13.4%.

The similarity in porosity values between the central axis (approximately 12–13%) and the gear teeth (approximately 12–13%) confirms that the radial pressure exerted by the elastic sleeve successfully mimicked isostatic pressing conditions, eliminating the density gradients typically associated with rigid dies.

### 3.3. Ejection Behavior

A definitive advantage of the proposed technology is the ejection mechanics. In all experimental trials, the spur gears were extracted from the die without the need for extraction force (Free Ejection). The theoretical mechanism was experimentally validated: the elastic relaxation (expansion) of the sleeve upon the removal of the pressing force was greater than the elastic spring-back of the metal powder compact. This differential relaxation caused the die walls to detach from the part surfaces, thereby eliminating friction-induced shear stresses and preventing the formation of laminar cracks or capping defects.

### 3.4. Simulation-Based Validation (FORGE)

To complement the experimental data, a 3D Finite Element Method (FEM) simulation was performed using FORGE software. The analysis focused on the strain distribution and the mechanical stress states within both the spur gear and the elastic die assembly during the compaction process.

#### 3.4.1. Strain Distribution (Equivalent Deformation)

**Figure 11:**
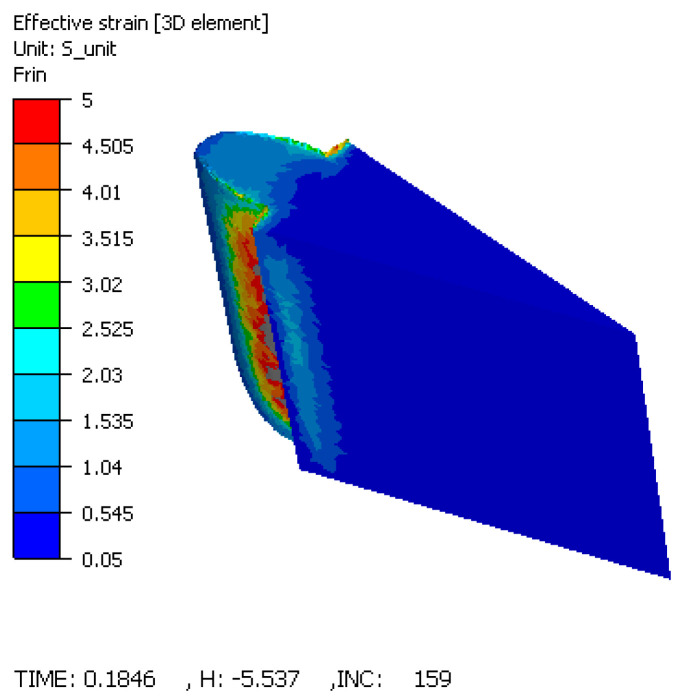
Equivalent strain distribution indicating maximum deformation at the tooth root.

The distribution of equivalent strain in the idealized solid model revealed the influence of the complex gear geometry and friction (Figure 11). Maximum strain values (4.5–5.0) were localized at the tooth root, while minimum values (1.04–1.53) occurred at the tooth tip. It should be emphasized that these values represent plastic deformation of a fully dense solid and do not directly correspond to powder densification strain. In actual powder compaction, these regions would experience particle rearrangement, pore collapse, and density gradients. However, the simulation provides qualitative insight into zones of high mechanical work [23,24], which correlate with the experimentally observed uniform porosity distribution.

#### 3.4.2. Stress Distribution (Von Mises)

**Figure 12:**
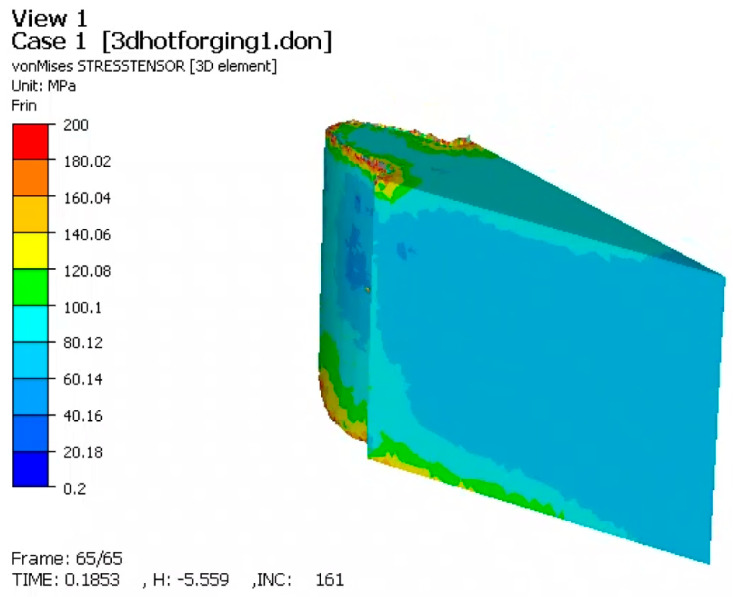
Von Mises stress distribution in the gear tooth flank.

The stress analysis provided crucial insights into both the quality of the part and the structural integrity of the tooling (Figure 12).

Stresses in the Gear: On the tooth flank, Von Mises stresses reached 180–200 MPa. The distribution was uniform across the height of the tooth, which suggests good dimensional stability and a low risk of residual stress-induced distortion after ejection.

**Figure 13:**
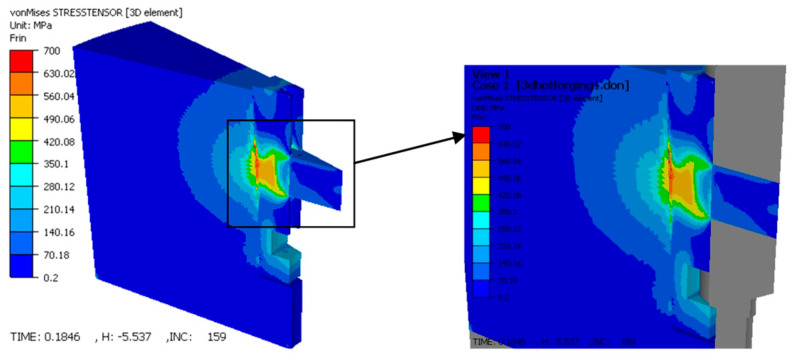
Von Mises stress distribution in the elastic sleeve and container at the end of deformation.

Stresses in the Tooling (Critical Interface): The simulation identified the interface between the elastic sleeve and the container as the most mechanically critical zone. As illustrated in Figure 13, stress in this region reached a maximum of 700 MPa.

This high-stress state is the result of the radial expansion of the sleeve against the rigid container. The cyclic nature of this loading (compression during pressing, relaxation during ejection) highlights this interface as the primary site for potential fatigue failure, necessitating the use of high-strength tool steels and careful pre-stressing calibration.

#### 3.4.3. Pressure Distribution

**Figure 14:**
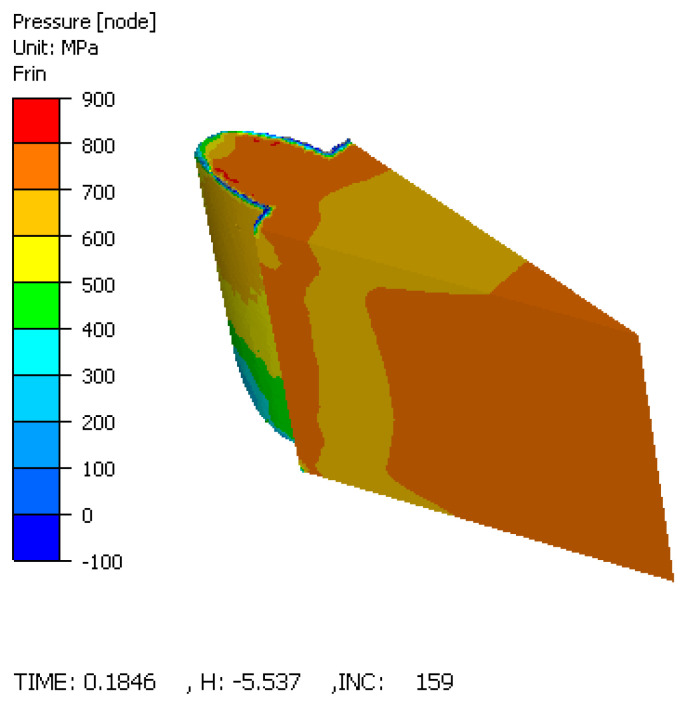
The pressure distribution showing peak values at the tooth root.

The forming pressure required to deform the material was found to be highest at the tooth base, reaching values of 700–800 MPa (Figure 14). On the tooth flank, the pressure varied between 500 and 800 MPa. These values validate the need for high-capacity presses and robust die construction when manufacturing complex gear geometries.

## 4. Discussion

### 4.1. Mechanisms of Densification

The experimental results utilizing Ancorsteel 2000 powder highlight distinct behaviors regarding lubricant efficiency and radial compression. At lower compaction pressures (400–500 MPa), the addition of lubricant (0.75% vs. 0.5%) played a significant role in particle rearrangement, yielding density improvements of nearly 10%. However, as pressure increased to 700 MPa, the influence of the lubricant diminished. This suggests that at high pressures, the process is dominated by the plastic deformation of the particles rather than inter-particle sliding, making the mechanical interlocking the primary densification mechanism.

Furthermore, the application of pre-stressing through the elastic sleeve stroke demonstrated a clear advantage over non-prestressed conditions. The radial pressure exerted by the sleeve effectively “squeezes” the powder perpendicular to the pressing axis. However, the marginal difference in sintered density between a 2 mm and a 3 mm stroke (approximately 3%) indicates a saturation point. Beyond a certain level of radial compression, the internal friction of the powder mass resists further densification, suggesting that an optimal stroke exists that balances density gain with energy input.

Based on the experimental data, the optimal combination of parameters to maximize the density (and thereby the structural integrity) of the Ancorsteel 2000 spur gears is a compaction pressure of 700 MPa, an elastic die pre-stressing stroke of 3 mm, and a lubricant content of 0.5 wt.%. This combination yielded the maximum sintered density of 7.51 g/cm^3^.

### 4.2. Structural Homogeneity and Isostatic Behavior

A persistent challenge in rigid die compaction of complex shapes, such as spur gears, is the “neutral zone” density dip, where friction prevents adequate pressure transmission. The microstructural analysis using ImageJ revealed a remarkable uniformity in porosity (approximately 12–13%) across both the central axis and the gear teeth. This uniformity confirms that the elastic die mimics isostatic pressing conditions. By compressing the powder radially (via the sleeve) simultaneously with the axial punch movement, the die minimizes the density gradients typically caused by wall friction [25,26]. This homogeneity is critical for the mechanical reliability of the gear, ensuring consistent fatigue strength across the tooth profile. While direct mechanical testing (e.g., tensile or gear tooth bending fatigue tests) was not performed in this current study, it is a fundamental principle of powder metallurgy that mechanical strength is directly correlated with final sintered density. Therefore, the parameters of the elastic die process that maximize density and minimize porosity variance proportionally increase the final strength of the manufactured gear. Specifically, by using the radial pre-stressing stroke to eliminate structurally weak ‘neutral zones’ and achieve uniform porosity, the elastic die technology ensures consistent load-bearing capacity and fatigue strength across the entire complex tooth profile.

### 4.3. Validation of Free Ejection

The complete elimination of extraction forces observed in all trials validates the theoretical model of the elastic die. The mechanism relies on differential elastic relaxation: the elastic expansion of the sleeve (ΔD) upon the removal of the pressing load is designed to be greater than the spring-back of the metal powder compact.

This separation effectively breaks the contact between the tool and the component before the vertical ejection stroke begins. Consequently, the common defects associated with ejection from rigid dies, such as laminar cracks, capping, and stick-slip induced surface roughness, are eliminated. This feature also implies a significant reduction in abrasive wear on the die walls, potentially extending tool life.

### 4.4. Tooling Integrity and Simulation Insights

While the experimental results confirm the quality of the part, the FORGE simulation provides crucial insights into the structural integrity of the tooling. The FEM analysis identified the interface between the elastic sleeve and the rigid container as a zone of high stress concentration, reaching values up to 700 MPa. Although the simulation models the powder as an equivalent solid (and therefore does not capture densification physics), it accurately predicts the mechanical loading on the tooling components. This stress arises from the sleeve expanding against the container walls during the compaction stroke. Given the cyclic nature of this loading (compression–relaxation cycles), this interface is susceptible to fatigue failure. The simulation suggests that while the part benefits from the elastic process, the tool design must incorporate high-strength materials or optimized taper angles (α) to withstand these localized stresses without plastic yielding or fatigue cracking.

It must be acknowledged that the discrepancy between the physical model (a porous, compressible powder mass) and the numerical simulation (an equivalent fully dense solid governed by the Norton–Hoff law) is a primary source of error regarding the internal strain distribution of the part. While the current model reliably predicts the mechanical load transferred to the tool interfaces, it cannot accurately map the kinematics of pore collapse. Therefore, necessary future validation will require coupling the elastic tool kinematics with advanced porous plasticity models (e.g., the Shima–Oyane or Drucker–Prager Cap models) to rigorously simulate the density evolution during the sleeve’s radial contraction.

### 4.5. Industrial Implications

The integration of these findings suggests that elastic die technology is a viable alternative for mass-producing high-density, complex gears. The ability to eliminate draft angles on the part allows the production of net-shape spur gears that require little to no secondary machining. However, the high stresses at the sleeve–container interface necessitate rigorous tool design to ensure that the benefits of reduced abrasive wear are not negated by fatigue failure of the die assembly.

Compared to conventional rigid die compaction, which necessitates substantial extraction forces that induce sliding friction wear, capping defects, and pronounced ‘neutral zone’ density gradients, the elastic die provides a clear operational advantage. The novel technology eliminates extraction friction and successfully achieves a homogenized, isostatic-like density distribution across complex gear profiles, effectively resolving the most persistent physical limitations of rigid tooling.

## 5. Conclusions

This research successfully validated the application of elastic die technology for the powder compaction of spur gears, offering a proven alternative to rigid die manufacturing. The following principal conclusions are drawn:

- *Operational Feasibility:* The proposed tool design, characterized by a mobile elastic sleeve, functioned correctly under high-pressure conditions (up to 700 MPa). The experimental trials confirmed that the radial deformation of the sleeve is controllable and reversible, allowing for the successful production of complex gear geometries without mechanical seizing.
- *Achievement of Free Ejection:* The most significant outcome is the experimental confirmation that extraction forces were effectively nullified. The elastic relaxation of the sleeve (ΔD) was sufficient to clear the gear teeth flanks, thereby producing components free of the laminar cracks and surface striations typically caused by ejection friction in rigid dies.
- *Homogeneity Verdict:* Quantitative image analysis established that the radial compaction pressure successfully counteracted the “neutral zone” effect. With porosity variances restricted to a narrow range (approx. 12–13%) across the entire gear cross-section, the technology is confirmed to produce parts with isotropic-like structural properties, superior to standard uniaxial compaction.
- *Simulation as a Design Tool:* The simulation successfully predicted critical stress concentrations of 700 MPa at the sleeve–container interface and provided qualitative insight into strain localization patterns. While the simulation does not quantitatively predict powder density (due to software limitations), the predicted high-strain zones at the tooth root correlate with the experimentally observed uniform densification. The simulation conclusively pinpointed the sleeve–container interface as the lifecycle bottleneck, dictating that future industrial implementations must prioritize high-fatigue-strength materials for these specific components.
- *Manufacturing Implications:* The study proves that elastic dies enable the manufacturing of net-shape gears with zero draft angles. By removing the geometric constraints required for ejection in rigid tools, this technology reduces the need for secondary machining operations and lowers the consumption of lubricants, presenting a viable pathway for cost-efficient, high-performance gear production.
- *Optimal Process Parameters:* The experimental results established that the highest densification for the manufactured spur gear is achieved using an optimal combination of 700 MPa compaction pressure, a 3 mm die pre-stressing stroke, and a 0.5 wt.% solid lubricant concentration. This specific parameter matrix yielded a maximum sintered density of 7.51 g/cm^3^, providing the best configuration for maximizing the mechanical strength of the component.

## Figures and Tables

**Table 1:** Distribution of finite element discretization network.

Component Type	Component Name	No. of Nodes	No. of Elements
Deformable bodies	Container	88,567	488,473
	Part	11,408	52,733
	Elastic sleeve	46,694	243,645
Rigid bodies	Upper Punch	16,311	32,618
	Lower Punch	11,423	22,842
	Die holder block	4984	9964

**Table 2:** Parameters of the model equation.

A—Consistency of the Solid [mm·Kg·s]	m_1_	m_2_	m_3_	m_4_
1498	0.002	0.126	0.145	0.059

**Table 3:** Experimental measurements of Green and Sintered Density (g/cm^3^) for Ancorsteel 2000 powder.

Compaction Pressure (MPa)	Die Stroke (mm)	Lubricant Content (%)	Green Density (g/cm^3^)	Sintered Density (g/cm^3^)
400	0	0.5	6.38	7.15
	2	0.5	6.57	7.20
	3	0.5	6.68	7.21
	0	0.75	6.17	7.10
	2	0.75	6.29	7.19
	3	0.75	6.53	7.34
500	0	0.5	6.96	7.23
	2	0.5	7.01	7.34
	3	0.5	7.09	7.39
	0	0.75	6.76	7.23
	2	0.75	6.87	7.28
	3	0.75	6.96	7.37
600	0	0.5	7.07	7.37
	2	0.5	7.17	7.45
	3	0.5	7.19	7.46
	0	0.75	7.03	7.30
	2	0.75	7.09	7.34
	3	0.75	7.16	7.41
700	0	0.5	7.21	7.46
	2	0.5	7.24	7.48
	3	0.5	7.26	7.51
	0	0.75	7.19	7.35
	2	0.75	7.20	7.39
	3	0.75	7.22	7.43

## Data Availability

The original contributions presented in this study are included in the article. Further inquiries can be directed to the corresponding author.
